# Immune regulation of a chronic bacteria infection and consequences for pathogen transmission

**DOI:** 10.1186/1471-2180-10-226

**Published:** 2010-08-25

**Authors:** Ashutosh K Pathak, Kathleen E Creppage, Jacob R Werner, Isabella M Cattadori

**Affiliations:** 1 Center for Infectious Disease Dynamics, Dept. Biology, The Pennsylvania State University, University Park PA 16802, USA; 2 Animal Resource Program, Centralized Biological Laboratory, The Pennsylvania State University, University Park PA 16802, USA

## Abstract

**Background:**

The role of host immunity has been recognized as not only playing a fundamental role in the interaction between the host and pathogen but also in influencing host infectiousness and the ability to shed pathogens. Despite the interest in this area of study, and the development of theoretical work on the immuno-epidemiology of infections, little is known about the immunological processes that influence pathogen shedding patterns.

**Results:**

We used the respiratory bacterium *Bordetella bronchiseptica *and its common natural host, the rabbit, to examine the intensity and duration of oro-nasal bacteria shedding in relation to changes in the level of serum antibodies, blood cells, cytokine expression and number of bacteria colonies in the respiratory tract. Findings show that infected rabbits shed *B. bronchiseptica *by contact up to 4.5 months post infection. Shedding was positively affected by number of bacteria in the nasal cavity (CFU/g) but negatively influenced by serum IgG, which also contributed to the initial reduction of bacteria in the nasal cavity. Three main patterns of shedding were identified: i- bacteria were shed intermittently (46% of individuals), ii- bacteria shedding fell with the progression of the infection (31%) and iii- individuals never shed bacteria despite being infected (23%). Differences in the initial number of bacteria shed between the first two groups were associated with differences in the level of serum antibodies and white blood cells. These results suggest that the immunological conditions at the early stage of the infection may play a role in modulating the long term dynamics of *B. bronchiseptica *shedding.

**Conclusions:**

We propose that IgG influences the threshold of bacteria in the oro-nasal cavity which then affects the intensity and duration of individual shedding. In addition, we suggest that a threshold level of infection is required for shedding, below this value individuals never shed bacteria despite being infected. The mechanisms regulating these interactions are still obscure and more studies are needed to understand the persistence of bacteria in the upper respiratory tract and the processes controlling the intensity and duration of shedding.

## Background

An appreciation of the immunological mechanisms that affect the interaction between the host and its pathogens is crucial for an understanding of the epidemiology of infection [1-4]. By linking within-host immunological processes to the between-host dynamics of infection it is possible to explain, and ultimately prevent, the conditions that allow for the invasion and survival of a pathogen within a host and the consequences for transmission. Fundamental to this is the knowledge of how the immune response affects pathogen replication and clearance as well as the intensity and duration of shedding and, thus, transmission.

Chronic bacteria infections can pose a challenge to the study of host infectiousness and associated immune response in that bacteria can either persist in the host, despite an acute inflammatory phase and active immunity, or colonize and persist without causing any apparent clinical or symptomatic effects [5-7]. Bacteria can activate their pathogenicity at a later time by triggering serious disease and high infectiousness or can increase their transmission rate in response to changes in host susceptibility [8-12]. These findings suggest that immune-compromised and chronically infected hosts can act either as life-long bacteria shedders or shed bacteria for a restricted period, usually coinciding with the acute phase of infection. To understand the dynamics of chronic infections, we need to identify not only the key immunological processes that affect long term pathogen persistence but also how pathogen replication, intensity and duration of bacteria shedding is associated with the immune response.

Here, we investigated the relationship between immune response and shedding rate in a chronic bacteria infection using the *Bordetella bronchiseptica*-rabbit system. Our recent work on the epidemiology of *B. bronchiseptica *in a free living population of rabbits (*Oryctolagus cuniculus*) showed that this is a common and persistent infection: annual prevalence ranged between 88% and 97% and by 2 months of age, 65% of the individuals had already seroconverted [13]. A model for bacteria infection was suggested where the annual recruitment of new infected individuals was associated with the onset of the host breeding season and the availability of new naïve offspring. Breeding, seropositive females represented the main source of infection for the newborns. However, it was not clear whether they were chronically infectious or in a re-activated infectious status due to the immuno-suppressed conditions during breeding.

Current knowledge on the immunology of *B. bronchiseptica *infection is largely derived from laboratory work with rats and mice and occasionally rabbits [14-21]. Studies on mice suggest that the bacterium stimulates an initial strong innate and subsequent acquired immune response characterized by the clearance of the bacteria from the lower respiratory tract but the persistence in the nasal cavity up to 270 days post infection, with the potential for life-long bacteria shedding [15]. The mechanisms involved in the persistence of bacteria in the nasal cavity are still unclear but the adhesin filamentous hemagglutinin (FHA) appears to play an important role in the colonization of the unciliated olfactory epithelia [22]. While highly informative, rats and mice show no documented ability for oro-nasal *B. bronchispetica *transmission and are not useful hosts for exploring the effect of host immunity on bacteria shedding and transmission in general [23,24].

Motivated by our recent work on the epidemiology of *B. brochiseptica *infection in a natural system, we examined whether chronically infected individuals can be long-term, constant bacteria shedders or whether the frequency and intensity of shedding changes with time and between individuals as constrained by their immune response; for example, hosts may not shed bacteria despite being chronically infected. We established a laboratory model system wherein rabbits were infected with *B. bronchiseptica *strain RB50 and acquired immunity and bacteria shedding was quantified for 150 days post infection. We focused our attention on the immunological parameters relevant to the dynamics of *B. bronchiseptica*, as previously identified in mice and rabbits, and examined how they affect the intensity and duration of the oro-nasal shedding.

## Results

To highlight heterogeneities in the shedding pattern and associated immune response between individuals, blood and tissue samples were individually processed.

### Infection of rabbits with *B. bronchiseptica *RB50

**Figure 1:**
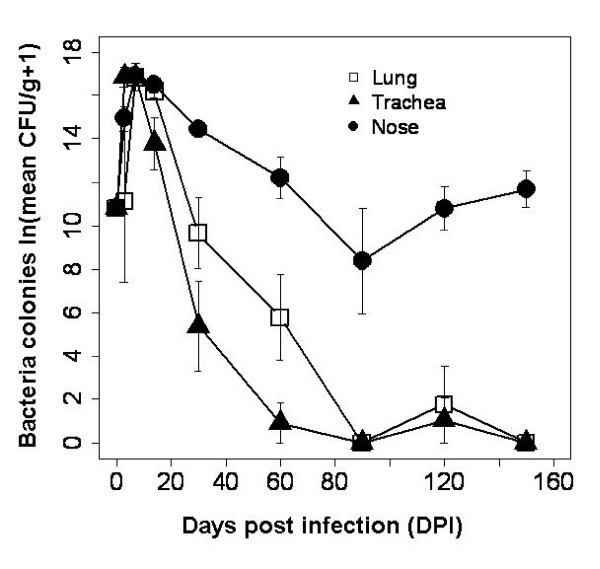
**Mean number of bacteria (CFUs/g ± S.E.) in the respiratory tract of infected rabbits at days 3, 7, 14, 30, 60, 90, 120 and 150 post-infection (DPI)**. Initial infection dose is reported (Day 0 = log(50,000 CFU/ml+1)). At each day post infection, lungs, trachea and nasal cavity were collected from 4 infected and 2 control rabbits and individually stored in PBS. Serial dilutions of the homogenates were plated out on BG blood agar plates supplemented with streptomycin. Bacteria were enumerated after incubating for 36-48hr at 37°C. The number of bacteria significantly declined with infection time (LME, DPI: P < 0.0001) and was significantly higher in the nares than trachea or lungs (LME, Organs: P < 0.0001).

Intranasal infection of rabbits led to the successful colonization and establishment of bacteria in the entire respiratory tract. By 3 days post infection (DPI) the mean number of bacteria colonies in the respiratory tract was 232 times higher than the initial inoculum (50,000 CFU/ml, Fig. 1). Levels peaked at day 7 post infection in all the three organs but quickly decreased thereafter and, by 150 days post infection, *B. bronchiseptica *was completely cleared from the trachea and lungs but persisted in the nares (Fig. 1). The number of bacteria consistently declined with the duration of the infection, DPI (coeff ± S.E.: -0.111 ± 0.013 d.f. = 30, P < 0.0001) but nares were significantly higher than either trachea or lungs (coeff ± S.E.: 0.069 ± 0.017 d.f. = 60 P < 0.0001), once differences among individuals and the non-independent sampling of the three organs from the same host were taken into account. Number of bacteria in the respiratory tract was negatively affected by serum IgG and circulating lymphocytes (coeff. ± S.E.: -6.5714 ± 1.002 and -0.853 ± 0.306, respectively) but positively influenced by circulating neutrophils (coeff. ± S.E.: 1.709 ± 0.524), when corrected by host variability and the non-independence of sampling the three respiratory organs from the same individual (For all: d.f. = 23, P < 0.01). The analysis repeated for each organ confirmed the negative effect of IgG on bacteria in the nares (coeff ± S.E.: -4.221 ± 0.854, d.f = 30, P < 0.0001) but also highlighted the positive effect of IL-10 (coeff ± S.E: -4.210 ± 0.512) and the negative role of IL-4 (coeff ± S.E: 3.431 ± 0.748) on bacteria in the lungs (analysis based on Ct values, for both: d.f. = 28, P < 0.0001). It is important to note that the cycle threshold (Ct) is inversely related to cytokine expression level, therefore and as reported above, the sign of the coefficient describing the CFU-Ct relationship should be interpreted as positive when negative and vice-versa. Results also showed a negative effect of serum antibodies and circulating lymphocytes (IgG, IgA and lymphocytes coeff ± S.E.: -9.564 ± 1.225, -5.046 ± 1.769 and -1.006 ± 0.372, respectively) and a positive effect of circulating neutrophils (coeff ± S.E.: 2.168 ± 0.636) on bacteria in the trachea (for all: d.f.= 22, P < 0.01). Overall, these findings support the hypothesis that IgG, IgA, neutrophils and lymphocytes are heavily involved in *B. bronchiseptica *clearance from the lower but not the upper respiratory tract, despite the negative effect of IgG. The positive association with neutrophils is probably caused by their rapid recruitment and short-lived contribution in the bacteria removal, as previously recorded [15,25]. Moreover, our results further support the suggestion of an immunological interference between antibody-mediated clearance (mainly by IgG) and antagonistic IL-10 anti-inflammatory activity in the lungs, which may explain the delay in bacteria clearance from this site as reported in other models [17].

### Bacteria shedding

**Figure 2:**
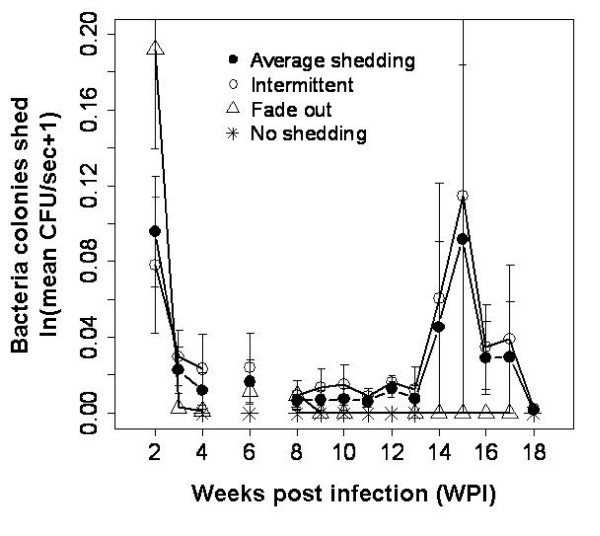
**Mean number of bacteria shed (CFUs/sec ± S.E.) by oro-nasal contact with a BG blood agar plate during the course of the infection**. A total of 14 infected rabbits were used and sacrificed at days 60, 90, 120 and 150 post-infection. Each individual was weekly challenged by oro-nasal contact with a BG blood agar plate and time of interaction measured. Bacteria were enumerated after incubating for 36-48 hr at 37°C. For every week post infection (from WPI 2 to WPI 18) the number of plates positive for *B. bronchiseptica *after removal of contaminated cases and sacrificed individuals was: WPI 2 = 8, WPI 3 = 6, WPI 4 = 8, WPI 5 = N.D. (no data), WPI 6 = 11, WPI 7 = N.D., WPI 8 = 14, WPI 9 = 12, WPI 10 = 12, WPI 11 = 12, WPI 12 = 12, WPI 13 = 8, WPI 14 = 8, WPI 15 = 8, WPI 16 = 8, WPI 17 = 8, WPI 18 = 4. The overall average shedding pattern and the more specific three shedding groups (intermittent, fade-out and non-shedding) are reported.

The goal of this study was to quantify the rate of *B. bronchiseptica *shedding in relation to the immune response and to use this finding to gain stronger insights into the epidemiology of a chronic infection. The strain of *B. bronchiseptica *used in this work was originally isolated from the nares of a 3 month old New Zealand White rabbit and it was assumed that it could be naturally transmitted between individuals [14]. Indeed, we found that rabbits were able to shed bacteria onto a BG blood agar plate by direct oro-nasal contact, which mimicked the natural contacts observed between free living individuals. Mean number of bacteria shed per second was 0.028 (± 0.001 S.E.) CFUs; shedding was high during the first month post infection and again 15 weeks later but substantially dropped between the two peaks (Fig. 2). Based on the longitudinal data (weekly sampling of individuals for serum antibodies and blood cells), we found a significant negative effect of IgG on number of bacteria shed (coeff ± S.E: -0.092 ± 0.025 df = 88 P < 0.0001), once corrected by host variability. Blood cells did not contribute to the pattern observed. The analysis was repeated using bacteria CFU counts from the nares of animals sampled at 60, 90, 120 and 150 post infection, and a weak but significant positive relationship was observed between bacteria shed at these sampling points and bacteria in the nasal cavity (coeff ± S.E.: 0.37e-7 ± 0.14e-7 d.f. = 8 P < 0.030). Together these results suggest that shedding is positively influenced by the level of infection in the oro-nasal cavity and negatively affected by serum IgG.

Three main patterns of shedding were identified during the course of the infection: i- bacteria were shed with variable intensities at irregular intervals ('intermittent' group, 46% of individuals), ii- intensity of bacteria shed fell with the progression of the infection ('fade-out' group, 31%) and iii- individuals never shed bacteria despite being infected ('non-shedders', 23%) (Fig. 2). To highlight immunological differences associated with these shedding patterns, analyses were repeated among the three groups using longitudinal blood data. No significant differences in serum IgG, IgA, neutrophils and lymphocytes were observed among the three patterns, however, the intercept of the models was consistently significant (for all: P < 0.05), once corrected for variability between hosts and their multiple sampling. This finding supports the hypothesis that the strength of the initial immune response is crucial in modulating the dynamics of shedding. During the second week post infection, differences in the dynamics of infection were observed between the intermittent and the fade-out group (no data were available for the non-shedding group). The relatively low number of bacteria shed by the intermittent group (mean CFU/sec. ± S.E.: 0.083 ± 0.019) was associated with low serum IgG (OD index ± S.E.: 0.238 ± 0.028) and high serum IgA (1.107 ± 0.052) as well as high circulating neutrophils (mean K/μL ± S.E.: 1.436 ± 0.158) and lymphocytes (mean K/μL ± S.E.: 2.150 ± 0.412). In contrast, the higher shedding in the fade out group (mean CFU/sec. ± S.E.: 0.213 ± 0.045) was correlated to high serum IgG (OD index ± S.E.: 0.434 ± 0.118) and low serum IgA (0.667 ± 0.128) and white blood cells (mean K/μL ± S.E., neutrophils: 0.896 ± 0.00 and lymphocytes: 0.740 ± 0.000). Although not conclusive or statistically significant, these relationships suggest that the strength of the early antibody and blood cells response may play a role in affecting both the initial and long-term pattern of *B. bronchiseptica *transmission.

### Host immune response overview

Overall, the immune response of rabbits to *B. bronchiseptica *infection confirmed previous findings reported in other animal models [14-19,25].

### Peripheral response

**Figure 3:**
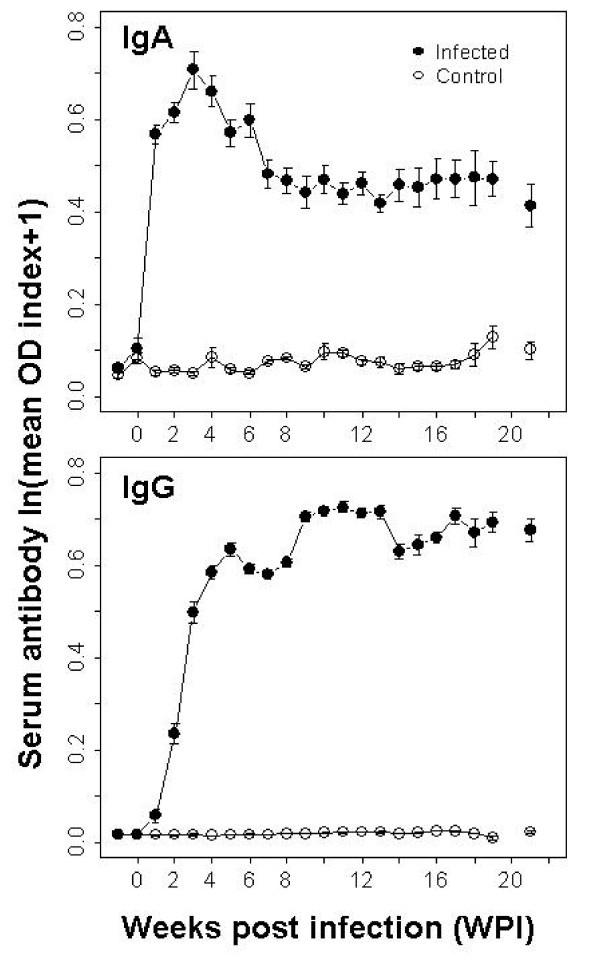
**Mean serum antibody response (OD index ± S.E.) in infected and control rabbits by sampling week (WPI)**. Serum was collected twice from all individuals prior to infection (48 rabbits sampled at week -1) and weekly thereafter. Number of samples decreased with time of infection as groups of 6 individuals (4 infected and 2 controls) were regularly sacrificed. Sera were assayed individually.

Infected hosts developed a strong serum IgG and IgA response compared to the controls (Fig. 3). The level of IgG rapidly increased in infected rabbits and remained consistently high for the duration of the infection, however and as previously highlighted, it was not sufficient to completely clear the bacteria from the upper respiratory tract (interaction between sampling time and infected-controls, coeff ± S.E.: 0.047 ± 0.005 d.f. = 328 P < 0.0001 -corrected for the random effect of the host and its longitudinal sampling). IgA levels in infected rabbits peaked around week three post infection and decreased thereafter, probably as a consequence of the successful clearance of bacteria from the lower respiratory tract [25,26]. Nevertheless, values remained significantly higher in infected compared to controls (coeff ± S.E.: 0.208 ± 0.056 d.f. = 45 P < 0.001) and for the duration of the experiment (interaction between infected-controls and sampling time, coeff ± S.E.: 0.0026 ± 0.001 d.f. = 410 P < 0.01; corrected for the host variability). Collectively, the systemic antibody profiles suggest that rabbit immune protection against *B. bronchiseptica *is robust for the first 5 months (150 days) post infection but does not result in complete clearance.

The neutrophil concentration in the blood decreased with the duration of the infection (coeff ± S.E.: -0.011 ± 0.002 d.f = 334 P < 0.0001) and was similar between infected and controls except in the first 2 weeks post-infection, where a significant neutrophilia was observed in infected compared to controls (coeff ± S.E.: 0.159 ± 0.075 d.f. = 27 P < 0.05). These findings further support the short-lived and early involvement of neutrophils in *B. bronchiseptica *clearance [15,27].

### Cytokine response in the lungs

**Figure 4:**
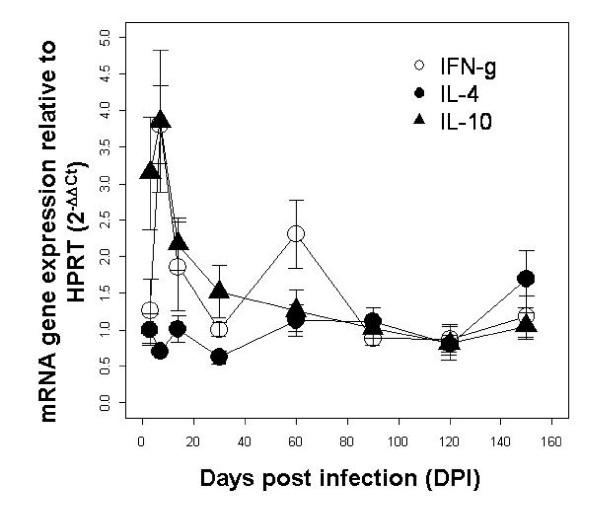
**Cytokine gene expression profiles in the lungs at days 3, 7, 14, 30, 60, 90, 120 and 150 post-infection (DPI)**. Cytokine data are presented using the 2^-ΔΔCt ^± S.E approach. Briefly, for each rabbit cytokine expression was scaled relative to the housekeeping gene HPRT (Ct), Ct values from infected individuals were then scaled over the controls.

As shown in fig. 4 and based on the 2^-ΔΔct ^transformation, a high IL-10 expression was observed in the lungs of infected rabbits in the first 30 days post infection, this was followed by a short-lived peak in IFN-γ at 60 days post infection, and a general decrease in cytokine expression thereafter. IL-4 showed consistent baseline expression. Overall and using the raw Ct values for analysis tractability, results confirmed the important anti-inflammatory role of IL-10 in *B. bronchiseptica *infected rabbits (interaction between infected-controls and sampling time, coeff ± S.E.: 0.001 ± 0.0001 d.f. = 41 P < 0.05, corrected for the random effect of the host). IFN-γ and IL-4 Ct values significantly changed among sampling time but not between infected and controls (respectively, coeff ± S.E.: 0.001 ± 0.0003 and -0.001 ± 0.0003 for both d.f. = 42 P < 0.05). Through its anti-inflammatory properties and involvement in the recruitment and activation of other anti-inflammatory cells [28,29], IL-10 probably facilitated the establishment of bacteria in the respiratory tract and the subsequent persistence in the nares, while the peaks at 7 and 60 days post infection in IFN-*γ *confirmed its important role in bacteria clearance from the lungs and possibly trachea. In summary, the dynamics of cytokine expression in the lungs of infected rabbits was in line with previous studies [20,21].

## Discussion

This study showed that rabbits infected with *Bordetella bronchiseptica *strain RB50 were able to shed bacteria by oro-nasal contact for at least 128 days post infection. Individuals naturally interacted with BG blood agar plates and a few minutes of direct contact were sufficient for the transmission of bacteria onto the medium. The mean number of bacteria shed followed the dynamics of infection, in that, shedding was high during the initial first month and decreased thereafter, although occasional peaks were observed up to 17 weeks post infection. The variability in the shedding pattern was unexpected but supports the hypothesis that rabbits with a chronic *B. bronchiseptica *infection can be long-term shedders, through a persistent infection in the upper respiratory tract. Specifically, most of the bacteria were shed at irregular intervals and with intensities that vary both within and between individuals. However, we also showed that some individuals never shed bacteria while infected, and this supports the hypothesis of a non-linear relationship between host infectiousness and *B. bronchiseptica *transmission. Moreover, since the immune system imposed constrains on the level and duration of infection we may argue that there was also a non-linear relationship between immune response and transmission dynamics.

The host acquired immunity, and probably the level of the early response, influenced the intensity, duration and pattern of bacteria shed. Serum IgG appeared to contribute to bacteria clearance in the lungs and trachea and the initial reduction in the nares. IgG also exerted a negative effect on the amount of *B. bronchiseptica *shed and together with IgA and white blood cells appeared to influence the initial and long-term shedding pattern. Indeed, a robust and timely IgG response probably modulated the long term shedding of *B. bronchiseptica *by quickly reducing or controlling replication in the nares below a threshold value required for consistent and prolonged pathogen transmission. In contrast, it is possible that the initial lower infection levels stimulated a milder immune response that allowed bacteria replication above a threshold necessary for long term shedding. While the number of bacteria in the nares was positively associated to the level of bacteria shed, some infected individuals never shed bacteria, supporting the hypothesis that a minimum threshold level of infection is necessary for bacteria shedding.

Serum IgA was probably more involved in the initial clearance of the lower respiratory tract, which agrees with the general role of this immunoglobulin in the early protection against invasive infections [26]. Serum IgG and IgA have been previously shown to be sufficient for *B. bronchiseptica *clearance in the lower but not the upper respiratory tract [16-18,25]. Similarly, neutrophils are involved in the early clearance of *B. bronchiseptica *from the lower respiratory tract [16,26,30]. Our findings on the role of serum antibodies and bacteria clearance are in line with previous work but also highlight the effect of serum IgG on the dynamics of *B. bronchiseptica *shedding.

At the cytokine level, the high IL-10 in the lungs during the initial 2 months of infection, was fundamental to delay clearance in the lower respiratory tract and probably contributed to the persistence of bacteria in the nasal cavity and associated shedding. Indeed, the absence of IL-10 synthesis has been related to augmented *B. bronchiseptica *clearance as well as reduced, albeit more effective, antibody production and higher IFN-γ in mice [17]. The association between serum antibodies, cytokines and bacteria shed has been reported in other host-bacteria systems. For example, a negative relationship between fecal shedding of *Escherichia coli *O157:H7 and IgG and IgA was observed in cows previously infected with a homologous bacteria strain [31]. Mucosal IgA was shown to reduce vaginal shedding and re-infection with *C. trachomatis *in mice [32], while human infections with *Campylobacter *spp. exhibited an inverse relationship between the shedding of fecal bacteria and age-dependent increases in serum IgG and IgA [33]. Moreover, IFN-*γ *expression appeared to contribute to the reduction of *Chlamydia trachomatis *and *C. muridarum *shedding in mice [34,35].

## Conclusions

We showed that rabbits were heterogeneous in their pattern of shedding *B. bronchiseptica *and that this was associated with differences in the host immune response. The dynamics of infection and partial clearance was consistent among individuals and a positive relationship was observed between bacteria shed and bacteria in the nasal cavity. Yet, some hosts shed bacteria intermittently, others shed bacteria only during the initial few weeks of infection while some individuals never shed bacteria. Together these findings suggest a strong non-linear relationship between force of infection, immune response and shedding rate for this chronic infection. The molecular mechanisms regulating these interactions are still obscure and more studies are needed to understand the persistence of bacteria in the upper respiratory tract as well as the processes controlling bacteria dispersal through direct oro-nasal contact or aerosol.

The occurrence of individuals that did not shed bacteria and the exclusion of a few contaminated plates, especially from the early part of the study, affected our search for a robust association between shedding patterns and the immune response. Nevertheless, the general patterns of bacteria dynamics and immune response, currently described, are consistent in this host-pathogen system as confirmed by our more recent studies on rabbits co-infected with *B. bronchiseptica *and gastrointestinal nematodes (unpubl. data). In conclusion, more attention should be given to the understanding of the relationship between host immune response, the level of infection and heterogeneities in pathogen shedding.

## Methods

### Bacteria strain and culture

The *Bordetella bronchiseptica *strain RB50 used in this study was kindly provided by Dr. E. T. Harvill (Penn State University, PA, USA). Bacteria were grown on Bordet-Gengou (BG) agar (HiMedia, USA) supplemented with 10% defibrinated sheep blood (Hema Resources, USA) and streptomycin (20 μg/ml, Sigma-Aldrich Co., USA). The bacteria inoculum was prepared by growing in Stainer-Scholte (SS) liquid culture medium at 37°C overnight on a rotary shaker to an optical density at 600 nm of approximately 0.3. For the infection, bacteria were re-suspended in sterile phosphate-buffered saline (PBS) at a density of 5 × 10^4 ^CFUs/ml, which was confirmed by plating serial dilutions of the inoculum on BG blood agar plates in triplicate.

### Study design

Out-bred, 60 days old New Zealand White male rabbits free from *B. bronchiseptica *and other pathogens/parasites (Harlan, USA), were housed in individual cages with food and water *ad libitum *and a 12 h day/night cycle. Individuals were lightly sedated intravenously with a pre-mixed solution of Ketamine (5 mg/kg, Phoenix Pharmaceuticals, USA) and Valium (0.25 mg/kg, Hospira, USA) and intra-nasally infected by pipetting in each nare 0.5 ml of PBS containing 2.5 × 10^4 ^*B. bronchiseptica *(dose adapted from [14]). Control animals were sham inoculated with 1 ml of sterile PBS. Groups of 6 individuals (4 infected and 2 controls) were euthanized with 1 ml of pentobarbital (Euthasol, Virbac) at days 3, 7, 14, 30, 60, 90, 120 and 150 post-infection (DPI) and the lungs, trachea and nasal cavity removed aseptically. Blood samples were collected weekly from the marginal ear vein of all animals. Animals were weighed weekly and monitored routinely for health status. All listed animal procedures were pre-approved by the Institutional Animal Care and Use Committee of The Pennsylvania State University.

### Quantification of bacteria in the respiratory tract

Following euthanasia, a weighed amount of the trachea and nasal cavity (turbinates and septum) were homogenized in 5 and 15 mls of PBS, respectively. The lungs were blended and approximately 3 g of the mix transferred into tubes containing RNAlater (Qiagen) and stored at -80°C for subsequent cytokine determination. The remaining tissue was homogenized in 15 ml of sterile PBS. Serial dilutions of the tissue homogenates were plated onto BG blood agar plates and incubated at 37°C for 48 hours to allow bacteria quantification.

### Quantification of bacteria shed

To monitor the weekly amount of bacteria shed, 14 infected rabbits were selected from the late sampling points (60, 90, 120 and 150 DPI). Every week, a BG blood agar plate was left in each cage for a maximum of 10 minutes and rabbits were allowed to interact with the plate by direct oral-nasal contact; the duration of each interaction was recorded. Plates were removed in case individuals chewed the plastic or ate the agar. Bacteria colonies were counted after incubation at 37°C for 48 hours; data were expressed as number of bacteria colonies shed per second of active interaction. This procedure is analogous to a natural transmission process compared to the nasal swabbing method that is more invasive, disruptive of the bacteria population and less representative of the individual's ability to shed bacteria [36-38]. We were not able to record the first week post infection data due to technical problems.

### Enzyme-Linked Immunosorbent Assay (ELISA)

Serum was collected once a week from all animals and separated from the red blood cells by centrifugation (10,000 rpm for 10 min) and stored at -80°C. Antigen coated plates were prepared by growing *B. bronchiseptica *overnight to mid-log phase in SS culture medium (OD at 600 nm of 0.6), washed once and re-suspended in PBS. Bacteria were heat inactivated at 65°C for 30 minutes, centrifuged at 5000 rpm for 15 minutes at 4°C and the resulting lysate estimated for protein concentration with the BCA assay (Pierce Biotechnology). The lysate was diluted in 0.2 M carbonate/bicarbonate coating buffer (pH 9.6) to obtain a final concentration of 6.5 μg/ml. 100 μl was used to coat the wells of 96-well polystyrene plates (Greiner Bio-One). Plates were incubated overnight at 4°C and then frozen at -20°C until use. Prior to serum addition, the plates were thawed at 37°C for 1 hour and blocked in 5% non-fat milk and PBS-T for 1 hour. The optimal serum dilution for the IgA and IgG ELISA assays was performed following Sanchez et al. [39] and Crowther [40]. A pool from strongly reacting serum samples (high pool prepared from infected individuals 4-6 weeks post-infection) and a pool from non-reacting serum samples (low pool from all individuals prior to infection) were prepared and a checkerboard titration was performed by serial dilutions of the strongly reacting serum pool against dilutions of the detection antibody, anti-rabbit IgA (Abcam, USA) or anti-rabbit IgG (Southern Biotechnology, USA). Optimal dilutions for the serum and detector antibody were selected by visually identifying the inflection point from the resulting dilution curves; the dilutions established for the serum were 1:10 for IgA and 1:10,000 for IgG, while for anti-rabbit IgA it was determined to be 1:5,000 and for anti-rabbit IgG, 1:10,000. Each sample from each individual was performed in duplicate with all plates having the high, low and background controls. Serum samples from each rabbit at every sampling point were added to the wells in blocking buffer at the appropriate final dilutions, and incubated at 37°C for 2 hours in a humidified chamber. Plates were then washed 4 times with PBS-T between each incubation and developed with 2,2'-Azino-bis(3-ethylbenzthiazoline-6-sulfonic acid) (Sigma-Aldrich) for 30 minutes and read with a spectrophotometer at 405 nm. Values were expressed as immunosorbent optical densities (OD).

To confirm the consistency of the ELISA results among plates, the relationship between corrected high antibody controls (high control - background control) and corrected low antibody controls (low control - background control) was examined; plates were repeated if the ratio was not consistent with a linear relationship among all plates showing a Pearson's correlation coefficient above r = 0.70. Background corrected antibody values were then transformed and standardized into optical density (OD) indexes as: Xi = (OD test sample-OD negative control)/(OD positive control-OD negative control) where Xi represents a replicate for each individual at every sampling point; the average of the two replicates (Xi = mean(X1+X2)) was then estimated for each individual at each sampling point [39]. To remove the occasional negative values, data were corrected as: Xi* = Xi+|min(total Xi)|. The new standardized mean optical density indexes were used in the statistical analyses.

### Haematology

A small aliquot (0.2 ml) of blood collected weekly from every animal was stored in EDTA coated tubes (Sartorius, Germany) and the most common cellular components in the blood (neutrophils, lymphocytes, monocytes, eosinophils, basophils and red blood cells) were quantified using the Hemavet 3 hematology system (Drew Scientific, USA).

### Quantification of cytokines gene expression in the lung

Cytokine gene expression in the lung was determined using a real-time quantitative PCR approach. For RNA extraction and purification, tissues were homogenized with a rotor-stator (Polytron PT 2100, Kinematica) in TRIzol reagent (Invitrogen). RNA was digested with 4 U TURBO DNA-free DNase (Ambion) and residual DNA contamination was assessed by performing quantitative PCRs. 1 μg of RNA was reverse transcribed with the High Capacity RNA-to-cDNA kit (Applied Biosystems). The resultant complementary DNA product (cDNA) was used to detect expression of IFN-γ, IL-4 and IL-10, with Hypoxanthine Phosphoribosyl Transferase (HPRT) as the housekeeping gene [41], using commercially available or custom made primers and probes (Applied Biosystems). The primer and probe sequences were based on a plasmid kindly provided by Dr. Sheila Lukehart (University of Washington, WA) [41]. The plasmid was also used to verify amplification efficiencies of the primer probe pairs (450 μM primers and 125 μM probe) by performing quantitative PCRs with 10-fold serial dilutions of the plasmid. The Pearson correlation coefficients between each cytokine and the housekeeping gene were always high (for all: r > 0.99). The sequences of the primer-probe pairs were as follows: HPRT (forward primer; 5'-CTCTCAACCTTAACTGGAAAGAATGTCT-3', reverse primer; 5'- GGAAAGCAAGGTCTGCATTGTT-3', probe 5'-6FAM-TTGCCAGTGTCAATTAT-NFQ-3'), IFN-γ (forward primer; 5'-GCTTTTCAGCTCTGCCTCATCTT-3', reverse primer; 5'- GGTTAGTGTGTCCTGGCAGTAA-3', probe 5'-6FAM-CAGCCGTAAGAACCC-NFQ-3'), IL-10 (Taqman assay ID; Oc03396942_m1, Applied Biosystems) and IL-4 (forward primer; 5'-ATGCACCAAGCTGATGATAGCA-3', reverse primer; 5'-CCTCTCTCTCGGTTGTGTTCTT-3', probe 5'-6FAM-CCCTGGCCGTCCCC-NFQ-3'). Quantitative PCRs were performed on an ABI7500 real time thermal cycler set in 'fast' mode for 40 cycles and using the PerfeCTa™ qPCR enzyme FastMix, UNG, Low ROX (Quanta Biosciences).

For visual purposes, changes in cytokines expression in the lungs were initially corrected for the house keeping gene (Ct values) and then scaled using the relative transcript level in fold change between infected at every sampling point (DPI) and the average of the controls from the whole experiment, following the comparative 2^-ΔΔCt ^method [42]. For analytical purposes, the corrected Ct values were used.

### Data analysis

Data were analyzed using linear mixed effect models (LME-REML) unless otherwise stated. To explore how bacteria shedding was affected by the host immune response, the number of colonies shed per interaction time was examined in relation to bacteria CFU count, antibody levels, blood cell values and infection time (week post infection WPI or days post infection DPI depending whether we used longitudinal or point based data). Individual identification code (ID) was considered as a random effect and the non-independent sampling of the same individual through time was quantified by including an autoregressive function of order 1 (AR1) on the individual ID. Changes in bacteria colonies established in the respiratory tract were examined in relation to the three respiratory organs and infection time (DPI), where individual ID was considered as a random effect and an autoregressive function of order 1 (AR1) was applied to the individual ID to take into account the non-independent response of the three correlated organs within each individual. This analysis was repeated for each organ and by including cytokines expression for the lungs. Linear mixed effect models were also performed to highlight differences between treatments (infected and control) and sampling time (WPI or DPI) in serum antibody response (IgA and IgG), white blood cells concentration and cytokine expression; again the individual ID was treated as a random or correlated effect (AR1) when necessary.

## Abbreviations

CFUs: Colony forming units; IgG: Immunoglobulin G; IgA: Immunoglobulin A; IL-10: Interleukin 10; IL-4: Interleukin 4; IFN-γ: Interferon gamma; FHA: filamentous haemagglutinin; DPI: Days post-infection; S.E.: Standard error; ID: Rabbit identification number; Ct: Cycle threshold; BG: Bordet-Gengou; OD: Optical density; SS: Stainer-Scholte; PBS: Phosphate buffered saline; PBS-T: PBS supplemented with 0.1% Tween-20; EDTA: Ethylene diamine tetraacetic acid; HPRT: Hypoxanthine Phosphoribosyl Transferase; ELISA: Enzyme Linked Immunosorbent Assay; AR1: Autoregressive function of order 1; LME-REML linear mixed model with restricted maximum likelihood.

## Authors' contributions

AKP performed the animal experiment, undertook the lab analysis and discussed with IMC the original idea and design of the experiments. KEC worked as an undergraduate research assistant and helped with the animal and lab work, JRW assisted during the lab experiment and undertook the hematological analysis and IMC designed the experiment, helped with the animal experiment, carried out the analysis and their interpretation, conceived the paper and the original study. IMC and AKP wrote the paper with critical comments from JRW. All authors approved the final manuscript.
